# An increased duplication of ZRS region that caused more than one supernumerary digits preaxial polydactyly in a large Chinese family

**DOI:** 10.1038/srep38500

**Published:** 2016-12-06

**Authors:** Bin Wang, Yutao Diao, Qiji Liu, Hongqiang An, Ruiping Ma, Guosheng Jiang, Nannan Lai, Ziwei Li, Xiaoxiao Zhu, Lin Zhao, Qiang Guo, Zhen Zhang, Rong Sun, Xia Li

**Affiliations:** 1 Department of peripheral vascular disease, Affiliated Hospital of Shandong University of Traditional Chinese Medicine, 42 Wenhua xi Road, Jinan 250011, Shandong, China; 2 Key Laboratory for Rare & Uncommon diseases of Shandong province, Institute of Basic Medicine, Shandong Academy of Medical Sciences, 18877 Jingshi Road, Jinan 250062, Shandong, China; 3 Department of medical genetics, Shandong University, School of Medicine, Jinan 250012, Shandong, China; 4 Department of Orthopedic Surgery, People’s Hospital of Xintai, Xintai 271200, Shandong, China; 5 Shandong Provincial Qianfoshan Hospital, 16766 Jingshi Road, Jinan 250014, Shandong, China; 6 Department of Immunology, School of Basic Medical Sciences, Fudan University, Shanghai 200032, China; 7 Shandong Academy of Chinese Medicine, Yanzi Shanxi Road, Jinan 250014, Shandong, China

## Abstract

Preaxial polydactyly (PPD) is inherited in an autosomal dominant fashion and characterized by the presence of one or more supernumerary digits on the thumb side. It had been identified that point mutation or genomic duplications of the long-range limb-specific cis-regulator - zone of polarizing activity regulatory sequence (ZRS) cause PPD or other limb deformities such as syndactyly type IV (SD4) and Triphalangeal thumb-polysyndactyly syndrome (TPTPS). Most previously reported cases involved with no more than one extra finger; however, the role of the point mutation or genomic duplications of ZRS in the case of more than one redundant finger polydactyly remains unclear. In this article, we reported a family case of more than one redundant finger polydactyly on the thumb side for bilateral hands with a pedigree chart of the family. Results of quantitative PCR (qPCR) and sequence analysis suggested that the relative copy number (RCN) of ZRS but not point mutation (including insertion and deletion) was involved in all affected individuals.

Polydactyly of the hand is a common congenital anomaly characterized by the presence of one or more supernumerary digits and, occasionally, extra metacarpals. In most cases, it is thought to occur as an autosomal-dominant trait with varied gene penetrance^1^. Polydactyly manifests itself varyingly in the number of extra fingers present as well as in the appearance of the affected fingers. Manifestations range from small cutaneous appendages to fully formed additional fingers. As for the Chinese population, reported cases usually occurred on the thumb side (preaxial polydactyly) or the ulnar side (postaxial polydactyly) and usually present unilaterally^2^.

A certain percentage of polydactyly cases involve gene mutations, but the exact chromosomal or genetic defects attributed to polydactyly have not yet been identified^3^. Previous research revealed that the mutations of *HOXD13* gene located on 2q31 can lead to syndactyly and (or) polydactyly malformation^4^,^5^,^6^,^7^,^8^. Subsequent studies demonstrated that the location that regulate human limb development was mapped to 7q36.3^9^,^10^,^11^, but the study failed to identify any corresponding mutations when analyzing corresponding candidate genes such as limb development membrane protein 1 (*LMBR1)*, motor neuron and pancreas homeobox 1 (*HLXB9)*, ring finger protein 32 (*RNF32)*, chromosome 7 open reading frame 13 (*C7ORF13)* and sonic hedgehog *(SHH)* in this locus^12^. Genomic duplications containing the zone of polarizing activity regulatory sequence (ZRS) were responsible for human SD4 and TPTPS in Chinese families^13^,^14^. As a gene regulatory sequence in intron 5 of *LMBR1*, its changes can affect the expression of important developmental genes such as *SHH*, a major determinant of the identity and numbers of digits in early limb development^15^.

In the present study, we reported an extremely rare family case that derived from a proband girl of polydactyly with more than one finger on the thumb side for bilateral hands. In addition to the surgical treatment of her hand anomaly, we described the components of this deformation in the family and pedigree background. Additionally, linkage analysis, gene sequencing and quantitative PCR (qPCR) analysis suggested that a higher duplication rather than mutations in the ZRS is associated with more than one finger PPD.

## Results

### Clinical report

**Figure 1:**
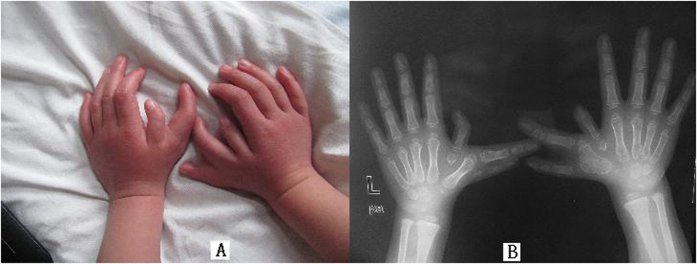
A view of both hands with 7 fingers of the five-year-old female proband (**A** Image of optical photograph; **B** Image of preoperative X-ray showed each hand has 2 triphalangeal thumbs).

The proband girl was found hand abnormality after her birth and diagnosed with polydactyly in both hands before the surgical treatment when she was 5 years old. Physical and X-ray imaging examination revealed multiple dysmorphisms of thumbs for both hands, i.e., both hands have three redundant and malformed thumbs (Fig. 1). No other congenital malformations were found and she was evaluated as being normal in both physiological and intellectual development.

The 3 months of follow-up after her surgical operation showed the girl’s recovery to be satisfactory. The surgical scars were faint, without obvious contraction and the girl’s parents were satisfied with the improved appearance and function of her hands (Supplementary Figure 1).

**Figure 2:**
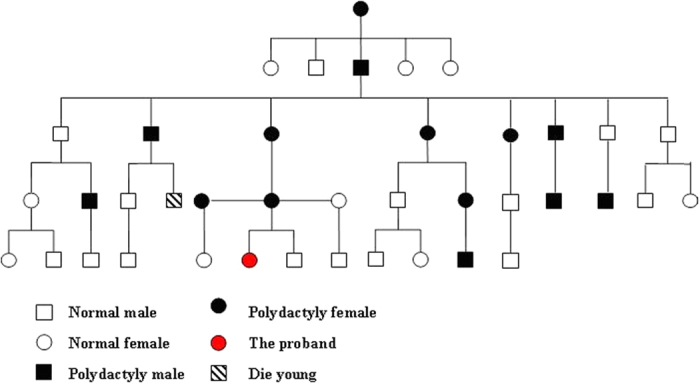
A pedigree chart of the 5 generation family with 24 normal and 16 polydactyly members that suggests an autosomal recessive inheritance. Squares and circles denote males and females respectively with filled ones as affected members. The proband was indicated by red and affected family members by black color and the square filled with slant lines in particular, represents the deceased family member.

The investigation of the family involved a total of 5 consanguineous generations composed of 40 immediate and collateral family members, with 16 relatives having polydactyly (8 males and 8 females). Thus the genetic type is elucidated as autosomal-dominant inheritance based on the pedigree chart (Fig. 2). The grandmother and most of her siblings suffered from deformities in both hands, and 7 out of 14 members in the mother’s generation presented with deformed thumbs. All 16 affected individuals were characterized by 2 or 3 extra fingers on the thumb side for both hands.

### Linkage analysis

**Figure 3:**
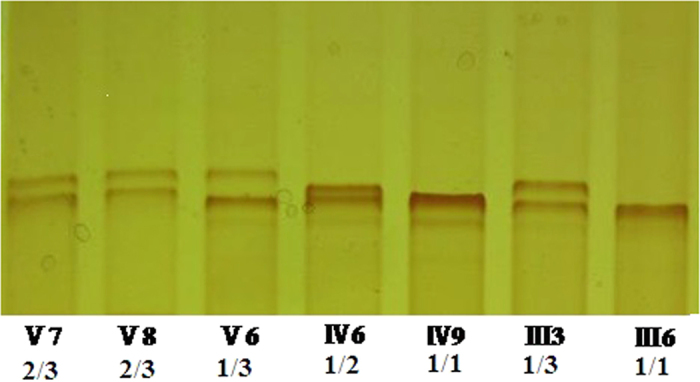
The silver staining of 8% denaturing polyacrylamide gel illustrated the genotypes of Microsatellite marker (short tandem repeat sequence, STR) P287. Lane 3–7 represent the 5 patients (V6, IV6, IV9, III3 and III6) who nexceptionally inherited allele 1 compared to the 2 normal family members (V7, V8) who have allele 2 and allele 3.

Linkage analysis and mutation detection were performed in this Chinese family with more than one extra finger PPD. Previous study had identified that P287 linked to 7q36.3 region tightly^9^; therefore, P287 was used as the typical genetic markers in Linkage analysis in this study. Figure 3 showed that the allelic separation of microsatellite marker P287 in 5 affected members in this family. All the 5 patients (V6, IV6, IV9, III3, III6) inherited allele 1 compared to the 2 unaffected family members (V7, V8), suggesting the phenomenon of gene and phenotype cosegregation. The data confirmed the association of the disease phenotype to chromosome 7q36.3. In other words, the finding suggests a possible linkage of finger deformity and Chr7q36.3 (especially ZRS) mutations.

### Variant detection

**Figure 4:**
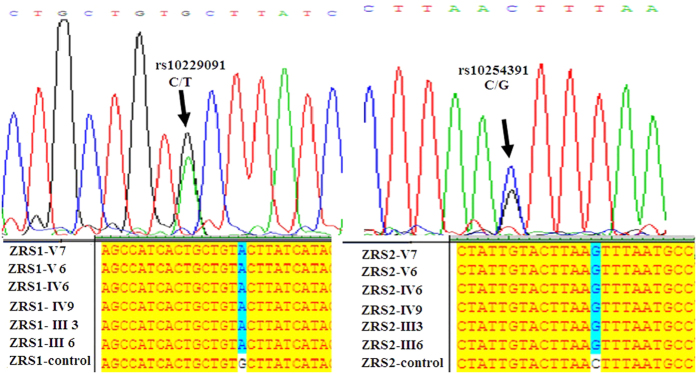
Identication of two single nucleotide polymorphisms (SNP) but not point mutations in 800 bp ZRS and the neighbouring region in the intron of *LMBR*1 gene. Sequences showing a heterozygous 156792210 C > T and a heterozygous 156791873 C > G polymorphic sites. Specifically, all the 6 family members have T and G alleles in rs10229091 and rs10254391 locus respectively, whereas the external control has C allele.

In order to perform mutation analysis on the approximately 800 bp ZRS of *LMBR1* intron 5, we designed 3 primer pairs (1F: GTGCCGAGATGCGGAAAT, 1R: TCTGGAGTGGAGGAGGGAG; 2F: TCTCCCTCCTCCACTCCA, 2R: TTGTTCCTCCTCTATTGTGCTGTCA; 3F: ACCAGGTGGAAGCGAAGA, 3R: ATACGCCCAGATTTGATG) to cover a 1833 bp region that contains ZRS. The data showed that a heterozygous C/T transition at position 4220 bp and a C/G transversion at 4758 bp of *LMBR1* were found in 6 family members compared with the external control (Fig. 4). However, the two point mutations had been identified to be common genetic polymorphism, but not the causations of limb deformities (http://www.ncbi.nlm.nih.gov/snp/?term=rs10229091; http://www.ncbi.nlm.nih.gov/snp/?term=rs10254391). No sequence alteration specific for PPD were found in the present study.

**Figure 5:**
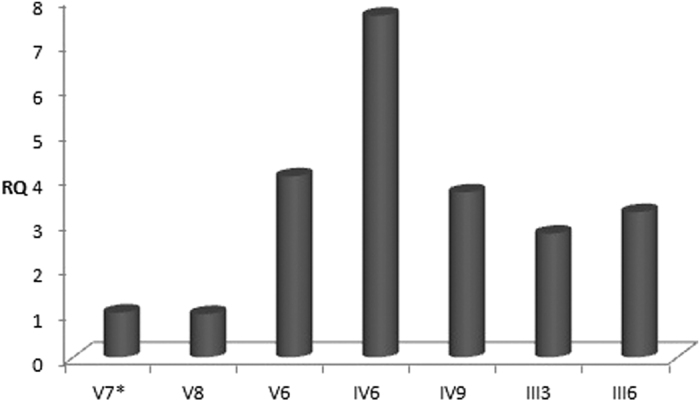
Duplication analysis of 726 bp fragment within ZRS region, where V7, V8, V6, IV6, IV9, III3 and III6 in the horizontal coordinate denote the family members corresponding to those in Fig. 3. In the vertical coordinate, we used RQ (relative quantity) as measurement unit and it is equivalent to relative copy number (RCN). V7 and V8 represent the unaffected family members whose RQ values are conspicuously lower than that of affected family members. *The absolute copy number of this unaffected family member acted as reference to calculate the value of RQ.

Detection of relative copy number (RCN) of the ZRS was based on the ^ΔΔ^C_T_ method. Samples of 5 affected individuals and 2 unaffected members in this family were obtained and the RCNs of almost more than 3-fold increase were detected in 5 affected individuals, while not in the unaffected family member (Table 1 and Fig. 5), indicating that the ZRS was duplicated or more probably, multiplicated in this case. These data confirmed the cosegregation of the copy number aberration and limb disorder with a full penetrance of the disorder in this family.

## Discussion

The limb defects resulted from a mutant ZRS showed a robust genotype-phenotype correlation but the phenotype presentation of digit abnormalities were defined with overlap. These digit deformities are approximately classified as preaxial polydactyly type II (PPD2), which includes isolated triphalangeal thumb (TPT), triphalangeal thumb-polysyndactyly syndrome (TPTPS), syndactyly type IV (SD4) and Werner mesomelic syndrome (WMS)^16^,^17^,^18^,^19^,^20^,^21^,^22^,^23^. One of the key genes that determines the correct limb development is *Shh*. In the early stage of embryonic development, the expression of *Shh* is restricted to the posterior margin of the limb bud as the zone of polarizing activity (ZPA) to ensure the function of expressed *Shh* to formulate the digit appearance and assign each digit to its correct position^12^,^13^,^14^. If *Shh* expresses in ectopic positions, i.e. the anterior end of the limb bud, one or more limb deformities may occur.

As a cis-regulatory element of 750–800 bp, ZRS is necessary and sufficient to regulate the activity of *Shh* both spatially and temporally, which in turn defines the ZPA^15^. Given that the ZRS is located inside intron 5 of the *Lmbr1* and a long distance (800kb-1Mb) upstream from its target position, the promoter of *Shh*, it is still an interesting question how the ZRS directs *Shh* expression to the ZPA. Genetic analysis has shown that the 5′HOXD factors, especially HOXD10 and 13 may bind to the ZRS for activation of *Shh* expression in the ZPA^24^,^25^. So long as *Shh* expression is triggered, a number of the ETS transcription factors, especially ETS1 and GABPa may set out to occupy at multiple sites within the ZRS to establish the boundary of *Shh* expression^26^. Two other ETS factors, the closely related ETV4 and ETV5, act to antagonise ETS1/GABPa activation. These ETS family members collaborate effectively to regulate *Shh* negatively in the anterior domain and positively in the posterior domain of ZPA^27^,^28^. ZRS point mutations can bring forth new, additional ETS1/GABPa binding sites, and the new binding sites are sufficient to overturn the inhibition of *Shh* expression and cause ectopic expression. Consequently, disregulation of *Shh* has relation to limb malformations^29^.

In this study, we reported a big family of 5 generations and 40 members with 16 family members have the appearance close to but different from the typical PPD or TPTPS. The affected family members have two superfluous triphalangeal thumbs without syndactyly and one biphalangeal thumb which were suspected to be the original thumb. We found that the copy number of ZRS region of affected family members was significantly higher than that of unaffected family members, but no point mutations in the ZRS region were found in these affected family members with this phenotype of two superfluous triphalangeal thumbs. These findings suggest that the interesting phenotype is hard to explain according to current genotype-phenotype mapping that only a point mutation of ZRS is sufficient to promote the expression of *Shh* in the anterior side of the limb bud and cause preaxial polydactyly, indicating the complexity of ZRS-associated human limb malformations. In addition to point mutation or small insertion/deletion within ZRS, the variants of ZRS also involve duplications of regions with varied length that contain ZRS and these duplications lead to a spectrum of outcomes ranging from TPTPS to SD4^11^,^23^,^30^,^31^. It has been suggested that this group of limb defects caused by point mutations within ZRS or duplications around ZRS should be collectively referred to as ‘ZRS-associated syndromes’^19^. However, quite a few unconventional phenotypes were also appeared when the ZRS duplication was present. Actually, all individuals surveyed in this study have no malformed toe despite the fact that previous report involved with the phenotype of both hand and foot deformities^31^. ZRS-associated human limb malformations should be considered as a continuum of phenotypes with varied limb appearance such as the affected individuals in this study carried the ZRS duplication but showed no syndactyly phenotype either in hands or in feet. Unfortunately, to date, no one-to-one mapping between the phenotype and genotype has been constructed.

Although no correlation between phenotype and point mutations in the ZRS region were founded in the affected family members in the present study, it was found that the copy number variation of ZRS region was significantly higher than that of the unaffected family members with the RCN fold increase from 2.76 to 7.63. Perhaps it is because of the greater copy number variation of ZRS duplication in this case compared with previously studies that exacerbates the phenotype of more than one triphalangeal thumbs. We speculated that the interaction between ZRS and *Shh* promoter may work by the topologically associating domains (TADs) ranging across the 800 kb-1 Mb distance in this case. This long distance enhancer (ZRS) - promoter contacts are formed via the TADs during limb differentiation, depending on the spatial structure of TAD and the available transcription factors acting on the locus. As shown in part A of Supplementary Figure 3, in normal condition, no particular three-dimensional (3D) structure in cells of the anterior margin of ZPA of limb bud is formed and the gene is inactive; therefore, the limb is developing normally. Conversely, certain degree of copy number variations with given length that contain ZRS, perhaps it happened to occur in this case, can firstly bring forth new 3D structure in cells of the ZPA anterior margin and then generates additional binding sites for transcription factors such as ETS1/GABPa, and the new binding sites are sufficient to overturn the inhibition of *Shh* expression and cause ectopic expression in the anterior domain of ZPA. This change may disturb the proper polarizing in ZPA and bring forth limb malformations (part B of Supplementary Figure 3). However, the scope of duplicated sequence flanking the core ZRS and its consequent 3D structure of TAD is unclear.

In short, we reported a large Chinese family suffered from more than one redundant triphalangeal thumbs in both hands that is discrepant from typical TPTPS or SD4. Results of quantitative PCR and sequence analysis indicated that the RCN of ZRS but not point mutation (insertion and/or deletion) is involved in all affected individuals. An intensive study such as microarray with much wider and higher throughput is needed to further identify the exact size of ZRS duplication and its regulative role for the formulation of digit anormaly. The findings will help to shed more light on the question that ‘ZRS-associated syndromes’ should be considered as a continuum of phenotypes.

## Materials and Methods

### Patient and ethical approval

A five-generation Chinese family in which fifteen members including the proband girl were affected by limb malformations of varied degree was investigated. All of the patients were mentally normal. Peripheral venous blood samples were collected from this girl and her 6 relatives in this family for subsequent genetic analysis. One sample from healthy individual out of this family was used as external control. This project was approved by the ethical committee of Shandong Academy of Medical Sciences (SAMS-128, 21-16-0930). All researchers and medical practitioners involved in this study have paid considerable attention to the dignity, rights, safety and well being of participants in accordance with the relevant guidelines of this committee. Written informed consent was obtained from the patient and participants in this study. All methods were performed in accordance with the relevant guidelines and regulations.

### Plastic surgery

A surgical operation was performed in order to reestablish the girl’s hand function. Firstly, the surgeons operated on the radial supernumerary thumb of left hand by means of a longitudinal incision of the skin, subcutaneous tissue and fascia in order to free and reveal the supernumerary finger of 3 phalanges. Flexor tendon attachment was seen and excised along with the supernumerary finger and the flexor tendon sutured to the radial side of the thumb, followed by dressing the skin edge and suturing the incision layer by layer. An S-shaped incision of skin, subcutaneous tissue and fascia was made on the kite side, followed by ligation of blood supply for the kite side supernumerary finger. The second supernumerary finger with 3 phalanges was freed, revealed and excised. Subsequently, the thumb metacarpophalangeal joint capsule was opened and tightly sutured. The skin edge was then repaired, and the incision was sutured sequentially (Supplementary Figure 2). Around the metacarpophalangeal joint capsule was tightly fastened by small splint for 1 month. This procedure was replicated on the right hand.

### Extraction of genome DNA

The genomic DNA was extracted from peripheral blood samples using a QIAamp Blood DNA Minikit (Qiagen GMBH, Hilden, Germany) according to the instruction. DNA sample out of this family was derived from a healthy volunteer. The quantity and quality of DNA were determined by using NanoDrop 1000 (Thermo, USA).

### Linkage analysis

Short tandem repeat (STR) sequence of P287 that tightly linked with chromosomal region 7q36.3 was used as genetic markers to explore whether these affected family members were regulated by genes (including *SHH* and *Lmbr*1) located in this region. The P287 amplicon was produced by using the primer pair of (F: TCCAGTCTGGGCAACAAGAGTG, R: GGAGGTTTTAGTGCTTCATCAG) to amplify a 255 bp PCR product followed by 8% denaturing polyacrylamide gel electrophoresis and silver staining to determine the cosegregation of limb deformity and P287 genotype. The amplification reactions were carried out in 0.2 mL PCR tubes using a thermocycler (Bio-Rad Laboratories). Each PCR reaction system consisted of 5.0 μL of 10 × buffer (25 mM MgCl_2_ contained), 0.5 μL dNTPs (10 mM each), 1 μL of primers (10 μM), 2.0 μL of the DNA sample, and sterile water to a total volume of 50 μL. PCR amplification program involved an initial DNA denaturation at 94 °C for 3 min, followed by 30 cycles of denaturation at 94 °C for 30 sec, annealing at 60 °C for 1 min and elongation at 72 °C for 1 min, which followed by a final extension at 72 °C for 7 min. The P287 genotypes were recognized according to the profile of electrophoresis. The bands that located in the bottom of the electrophoresis pattern represented the longest allele which is defined as allele 1 whereas the bands of second long defined as allele 2, and so on.

### Mutant detection

PCR and DNA sequencing method were used to detect the genetic mutations within ZRS. Three amplicons covering this region and its two side flanks were produced by polymerase chain reaction. The PCR reaction mixture and thermo cycle were consistent with those of linkage analysis described above. The amplified products were purified from agarose gel using a QIA quick Gel Extraction Kit (Qiagen GMBH, Hilden, Germany) and sequenced via the ABI3730XL sequencer (Applied Biosystems, Foster City, CA, U.S.).

### Real-time quantitative PCR

The real-time quantitative PCR (qPCR) was performed to confirm ZRS duplication based on the ^ΔΔ^C_T_ method. A 773 bp amplicon within ZRS was designed (F: GATTTGAAGTCATAGCATAAAA, R: TTGGGAAAATCAAATTAACAC). The qPCR was performed in a total volume of 20 ul in each tube containing 10 ul of SYBR premix Ex Tap (TaKaRa), 3 ul of genomic DNA, 1 ul of primers, and 8 ul nuclease-free water.

Reactions were run in Applied Biosystems 7500 Instrument (Hercules, California, USA). For qPCR, the reaction was initiated at 95 °C for 3 min for initial template denaturation, followed by a cycling protocol (95 °C for 15 s, 62 °C for 15 s, and 72 °C for 20 s for 30 cycles and a final elongation (72 °C for 1 min). After the final cycle, melt curve analysis was performed to confirm the amplification specificity. Date analysis was performed using the software accompanied with Applied Biosystems 7500 Instrument. All samples were assayed in triplicates.

## Additional Information

**How to cite this article**: Wang, B. *et al*. An increased duplication of ZRS region that caused more than one supernumerary digits preaxial polydactyly in a large Chinese family. *Sci. Rep.* **6**, 38500; doi: 10.1038/srep38500 (2016).

**Publisher's note:** Springer Nature remains neutral with regard to jurisdictional claims in published maps and institutional affiliations.

## Supplementary Material

Supplementary Information

## Figures and Tables

**Table 1:** Duplication analysis of 726 bp fragment within ZRS region.

Sample	C_T_ (Mean)	C_T_ (Std Dev)	^Δ^C_T_ (Mean)	^Δ^C_T_ (Std Err)	^ΔΔ^C_T_	RQ	RQ (Min)	RQ (Max)
V7*	36.4793	1.1429	17.2882	0.6654	0.0000	1.0000	0.2779	3.5989
V8	37.2986	1.0209	16.8888	0.6036	−2.1623	0.9675	0.2573	3.3686
V6	32.5860	0.3316	15.2731	0.3931	−2.0151	4.0420	1.8971	8.6120
IV6	32.9437	0.9258	14.3565	0.6409	−2.9317	7.6299	2.2224	26.1942
IV9	32.7875	0.3269	15.4066	0.2396	−1.8816	3.6849	2.3238	5.8432
III3	33.0184	0.1990	15.8218	0.1169	−1.4664	2.7633	2.2065	3.4605
III6	32.9968	0.2862	15.5906	0.1712	−1.6976	3.2436	2.3329	4.5098

RQ denotes relative quantity and was used here as the equivalent to relative copy number (RCN).

^*^The unaffected family member acted as referential calibrator.

## References

[b1] Koçer U., Aksoy H. M., Tiftikcioğlu Y. O. & Karaaslan O. Polydactyly: a study of four generations of a Turkish family including an affected member with bilateral cleft lip and palate. Scandinavian Journal of Plastic & Reconstructive Surgery & Hand Surgery 36, 284–288 (2002). 12477087 10.1080/028443102320791833

[b2] Yu X. L., Liu H. F. & Chen Q. Classification and Treatment for Congenital Polydactyly Deformity. Chinese Journal of Pediatric Surger. 20, 301–302 (in Chinese) (1999).

[b3] Hu J. & He L. Patterning mechanisms controlling digit development. Journal of genetics and genomics 35, 517–524 (2008). 18804070 10.1016/S1673-8527(08)60071-5

[b4] Muragaki Y., Mundlos S., Upton J. & Olsen B. R. Altered growth and branching patterns in synpolydactyly caused by mutations in HOXD13. Science 272, 548–551 (1996). 8614804 10.1126/science.272.5261.548

[b5] Sarfarazi M., Akarsu A. N. & Sayli B. S. Localization of the syndactyly type II (synpolydactyly) locus to 2q31 region and identification of tight linkage to HOXD8 intragenic marker. Human molecular genetics 4, 1453–1458 (1995). 7581388 10.1093/hmg/4.8.1453

[b6] Goodman F. R. & Scambler P. J. Human HOX gene mutations. Clinical genetics 59, 1–11 (2001). 11206481 10.1034/j.1399-0004.2001.590101.x

[b7] Dai L. . Mutation analysis of HOXD13 gene in a Chinese pedigree with synpolydactyly. Chinese journal of medical genetics 22, 277–280 (2005). 15952114

[b8] Zhao X. L. . HOXD13 polyalanine tract expansion in synpolydactyly: mutation detection and prenatal diagnosis in a large Chinese family. Chinese journal of medical genetics 22, 5–9 (2005). 15696469

[b9] O T. . A complex bilateral polysyndactyly disease locus maps to chromosome 7q36. Nature genetics 6, 282–286 (1994). 8012391 10.1038/ng0394-282

[b10] Heutink P. . The gene for triphalangeal thumb maps to the subtelomeric region of chromosome 7q. Nature genetics 6, 287–292 (1994). 8012392 10.1038/ng0394-287

[b11] Sevim B. . Phenotypic variability of triphalangeal thumb-polysyndactyly syndrome linked to chromosome 7q36. American journal of medical genetics 87, 399–406 (1999). 10594878

[b12] Sato D. . A syndactyly type IV locus maps to 7q36. Journal of human genetics 52, 561–564 (2007). 17476456 10.1007/s10038-007-0150-5

[b13] Sun M. . Triphalangeal thumb-polysyndactyly syndrome and syndactyly type IV are caused by genomic duplications involving the long range, limb-specific SHH enhancer. Journal of medical genetics 45, 589–595 (2008). 18417549 10.1136/jmg.2008.057646

[b14] Wu L. . A ZRS duplication causes syndactyly type IV with tibial hypoplasia. American journal of medical genetics. Part A 149A, 816–818 (2009). 19291772 10.1002/ajmg.a.32740

[b15] VanderMeer J. E. & Ahituv N. cis-regulatory mutations are a genetic cause of human limb malformations. Developmental dynamics : an official publication of the American Association of Anatomists 240, 920–930 (2011). 21509892 10.1002/dvdy.22535 PMC3174732

[b16] Lettice L. A. . Opposing functions of the ETS factor family define Shh spatial expression in limb buds and underlie polydactyly. Developmental cell 22, 459–467 (2012). 22340503 10.1016/j.devcel.2011.12.010 PMC3314984

[b17] Mao J. . Fgf-Dependent Etv4/5 Activity Is Required for Posterior Restriction of Sonic hedgehog and Promoting Outgrowth of the Vertebrate Limb. Developmental cell 16, 600–606 (2009). 19386268 10.1016/j.devcel.2009.02.005 PMC3164484

[b18] Zhang Z., Verheyden J. M., Hassell J. A. & Sun X. FGF-regulated Etv genes are essential for repressing Shh expression in mouse limb buds. Developmental cell 16, 607–613 (2009). 19386269 10.1016/j.devcel.2009.02.008 PMC3541528

[b19] Albuisson J. . Identification of two novel mutations in Shh long-range regulator associated with familial pre-axial polydactyly. Clinical genetics 79, 371–377 (2011). 20569257 10.1111/j.1399-0004.2010.01465.x

[b20] Gurnett C. A. . Two novel point mutations in the long-range SHH enhancer in three families with triphalangeal thumb and preaxial polydactyly. American journal of medical genetics. Part A 143A, 27–32 (2007). 17152067 10.1002/ajmg.a.31563

[b21] Klopocki E. . A microduplication of the long range SHH limb regulator (ZRS) is associated with triphalangeal thumb-polysyndactyly syndrome. Journal of medical genetics 45, 370–375 (2008). 18178630 10.1136/jmg.2007.055699

[b22] Sun M. . Triphalangeal thumb-polysyndactyly syndrome and syndactyly type IV are caused by genomic duplications involving the long range, limb-specific SHH enhancer. Journal of medical genetics 45, 589–595 (2008). 18417549 10.1136/jmg.2008.057646

[b23] Furniss D. . A variant in the sonic hedgehog regulatory sequence (ZRS) is associated with triphalangeal thumb and deregulates expression in the developing limb. Human molecular genetics 17, 2417–2423 (2008). 18463159 10.1093/hmg/ddn141 PMC2486440

[b24] Hill R. E. How to make a zone of polarizing activity: Insights into limb development via the abnormality preaxial polydactyly. Development Growth & Regeneration 49, 439–448 (2007). 10.1111/j.1440-169X.2007.00943.x 17661738

[b25] Zhu J. . Uncoupling Sonic hedgehog control of pattern and expansion of the developing limb bud. Developmental cell 14, 624–632 (2008). 18410737 10.1016/j.devcel.2008.01.008 PMC8284562

[b26] Towers M., Mahood R., Yin Y. & Tickle C. Integration of growth and specification in chick wing digit-patterning. Nature 452, 882–886 (2008). 18354396 10.1038/nature06718

[b27] Lettice L. A. . A long-range Shh enhancer regulates expression in the developing limb and fin and is associated with preaxial polydactyly. Human molecular genetics 12, 1725–1735 (2003). 12837695 10.1093/hmg/ddg180

[b28] Tarchini B. & Duboule D. Control of Hoxd genes’ collinearity during early limb development. Developmental cell 10, 93–103 (2006). 16399081 10.1016/j.devcel.2005.11.014

[b29] Capellini T. D. . Pbx1/Pbx2 requirement for distal limb patterning is mediated by the hierarchical control of Hox gene spatial distribution and Shh expression. Development 133, 2263–2273 (2006). 16672333 10.1242/dev.02395

[b30] M F. . Preaxial polydactyly/triphalangeal thumb is associated with changed transcription factor-binding affinity in a family with a novel point mutation in the long-range cis-regulatory element ZRS. European Journal of Human Genetics Ejhg 18, 733–736 (2010). 20068592 10.1038/ejhg.2009.225 PMC2987342

[b31] Semerci C. N. . Homozygous feature of isolated triphalangeal thumb–preaxial polydactyly linked to 7q36: no phenotypic difference between homozygotes and heterozygotes. Clinical genetics 76, 85–90 (2009). 19519794 10.1111/j.1399-0004.2009.01192.x

