# Supplementary material for: An increased duplication of ZRS region that caused more than one supernumerary digits preaxial polydactyly in a large Chinese family
Source: Sci Rep. 2016 Dec 6;6:38500. doi: 10.1038/srep38500 (PMC5138840; doi:10.1038/srep38500)
Supplement: Supplementary Information [file srep38500-s1.pdf]

An increased duplication of ZRS region that caused more than one supernumerary digits preaxial polydactyly in a large Chinese family

Bin Wang[1▲], Yutao Diao[2▲], Qiji Liu[3], Hongqiang An[4], Ruiping Ma[5],Guosheng Jiang[2], Nannan Lai[6,] Ziwei Li[2], Xiaoxiao Zhu[2], Lin Zhao[2], Qiang Guo[2], Zhen Zhang[2], Rong Sun[7], Xia Li[2*]

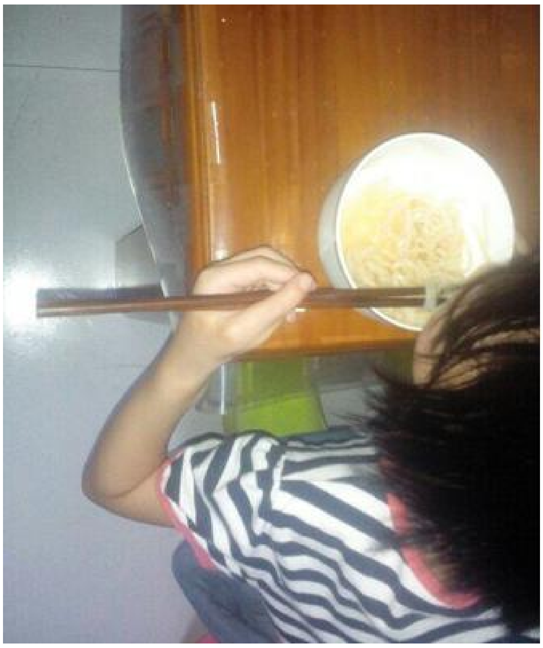

Supplementary Figure 1. This scene showed the recovery of the left hand function 3 months after the surgery.

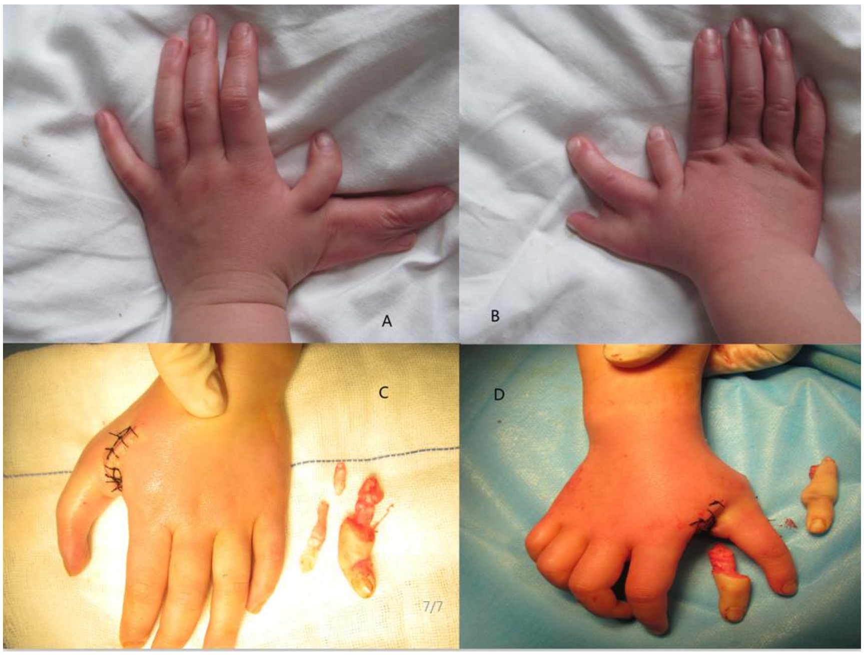

Supplementary Figure 2. The view just before and after the plastic surgery showed the radial

second triphalangeal thumb was retained and adapted to serve as the originally normal thumb. (A: Left hand before operation; B: Right hand before operation; C: Left hand after operation; D: Right hand after operation).

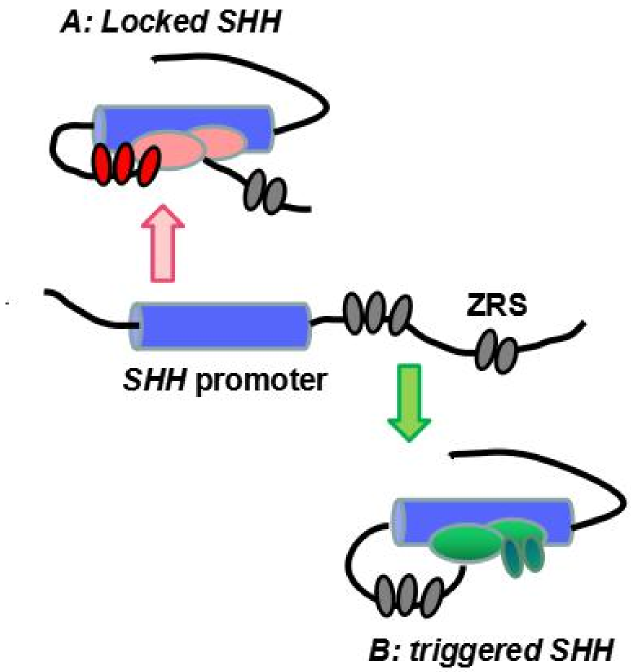

Supplementary Figure 3. ZRS-SHH promoter contact in ZPA.
